# Enterocutaneous fistulas: a primer for radiologists with emphasis on CT and MRI

**DOI:** 10.1007/s13244-017-0572-3

**Published:** 2017-09-29

**Authors:** Massimo Tonolini, Paolo Magistrelli

**Affiliations:** Department of Radiology, “Luigi Sacco” University Hospital, Via G.B. Grassi 74, 20157 Milan, Italy

**Keywords:** Enterocutaneous fistula, Malignant fistula, Crohn’s disease, Computed tomography (CT), Magnetic resonance imaging (MRI)

## Abstract

**Abstract:**

Enterocutaneous fistulas (ECFs) represent abnormal communications between the gastrointestinal tract and the skin. Nowadays, the majority (~80%) of ECFs develops secondary to abdominal surgeries; alternative, less common causes include chronic inflammatory bowel diseases (IBD) such as Crohn’s disease, tumours, and radiation enteritis in descending order of frequency. These rare disorders require thorough patient assessment and multidisciplinary management to limit the associated morbidity and mortality. This pictorial review includes an overview of causes, clinical manifestations, complications and management of ECFs. Afterwards, the imaging appearances, differential diagnoses, and therapeutic options of post-surgical, IBD-related, and malignant ECFs are presented with case examples. Most of the emphasis is placed on the current pivotal role of CT and MRI, which comprehensively depict ECFs providing cross-sectional information on the underlying postsurgical, neoplastic, infectious, or inflammatory conditions. Radiographic fistulography remains a valid technique, which rapidly depicts the ECF anatomy and confirms communication with the bowel. The aim of this paper is to increase radiologists’ familiarity with ECF imaging, thus allowing an appropriate choice between medical, interventional, or surgical treatment, ultimately resulting in higher likelihood of therapeutic success.

**Teaching Points:**

• *Enterocutaneous fistulas may complicate abdominal surgery, sometimes Crohn’s disease and tumours*.

• *The high associated morbidity and mortality result from sepsis, malnutrition and metabolic imbalance*.

• *The multidisciplinary management of ECFs requires thorough imaging for correct therapeutic choice*.

• *Radiographic fistulography rapidly depicts fistulas and communicating bowel loops in real-time*.

• *Multidetector CT and MRI provide cross-sectional information on fistulas and underlying diseases*.

## Introduction

Enterocutaneous fistulas (ECFs) are defined as abnormal communications between the gastrointestinal (GI) tract and the skin. Albeit relatively rare compared to past decades, ECFs still represent one of the most challenging conditions encountered in surgical practice, burdened with severe morbidity, impaired quality of life, and substantial mortality [1–3].

In recent years, specialised centres developed a robust management approach, which combines experience from surgeons, interventional radiologists, intensive care physicians, nutritionists, wound care specialists and nurses. Summarised in Table 1, this multidisciplinary treatment relies on thorough diagnostic imaging for correct patient selection and therapeutic choice: unfortunately, very limited literature exists on cross-sectional imaging of ECFs [4, 5].

**Table 1:** Fundamentals of the current multispecialist approach to enterocutaneous fistula management

Step no.	Treatment basics	Comments
1	Control of sepsis	Often requiring intensive care support Imaging-guided drainage of abscess collections
2	Limiting output volume	Octreotide administration Bowel rest, enteral, or parenteral nutrition
3	Wound or skin care	Use of dressings or bags depending on output Suction or Vacuum-Assisted Closure (VAC) devices if available
4	Metabolic and nutritional optimisation	Adequate hydration, electrolytes balance, nutritional status Proximal versus location in the bowel influences nutritional and fluid requirements
5	Assessing likelihood of spontaneous closure	Versus percutaneous treatment or elective surgical repair Factors associated with favourable healing: - narrow-calibre and/or relatively long (>2 cm) ECFs, - small enteric defect or anastomotic dehiscence (<1 cm) Factors associated with probable non-healing: - presence of foreign bodies, - history of irradiation, - active infection, - untreated chronic inflammatory bowel disease, - untreated tumours, - distal obstruction

This pictorial review relies upon experience at a tertiary referral hospital where oncologic and chronic inflammatory bowel disease (IBD) surgeries are performed. It includes an overview of causes, clinical manifestations, complications, and management of ECFs. Afterwards, the imaging appearances, differential diagnoses and therapeutic options of post-surgical, chronic inflammatory bowel diseases (IBD)-related and malignant ECFs are presented with examples. Most of the emphasis is placed on the current pivotal role of CT and MRI, which comprehensively depict ECFs providing cross-sectional information on the underlying postsurgical, neoplastic, infectious, or inflammatory disorders.

Our aim is to increase radiologists’ familiarity with ECF imaging, thus allowing an appropriate choice between medical, interventional, or surgical treatment, ultimately resulting in higher likelihood of therapeutic success.

## Clinical overview of enterocutaneous fistulas

The origin of an ECF may lie anywhere along the GI tract, and is found in the small bowel, colon, stomach, and duodenum in descending order of frequency. Nowadays, the majority (75–85%) of ECFs develop postoperatively following oncologic abdominal surgery, bowel resections for Crohn’s disease (CD), ventral hernioplasty, and repeated laparotomies. The remaining, spontaneous ECFs occur secondary to IBD (mostly CD rather than indeterminate and ulcerative colitis), fistulising tumours, radiation enteritis, colonic diverticulitis, intra-abdominal sepsis, and trauma, in descending order of frequency [1–3, 6].

The hallmark clinical finding is the external fistulous opening (EFO) at the anterior or lateral abdominal wall, which is often surrounded by an inflamed, tender cutaneous region, and may sometimes be hidden beneath a skin fold, or lie in the site of a recent or still patent surgical incision. Discharge may be either intermittent or continuous, sometimes purulent or faecal. The ECF output may be quantified and categorised as low (below 200 mL/day), moderate or high (over 500 mL/day). The macroscopic and biochemical features of the effluent provide useful clues to the ECF origin [1–3, 6].

Historically, the ECF-related mortality was in excess of 50%, particularly in those patients with high-output fistulas, tumours and sepsis. During the last 15 years, the improvements in surgery, wound care, nutritional and metabolic support resulted in decreased fatality rates (8–13%) and ultimate therapeutic success after a median 6 weeks in 75–91% of patients [1–3, 6].

However, rates of nonoperative healing remain low (~20–27% of cases overall) and ECFs still represent chronic, debilitating conditions associated with prolonged intensive care unit and hospital stays. The ECF-associated morbidity arises from sepsis, malnutrition, and metabolic imbalance. The invariable fluid and electrolyte abnormalities result from the combined effect of protracted catabolic febrile state, ileus or obstruction, loss of bowel integrity, and absorption. Further complications include generalised peritonitis, abdominal wall abscesses, and necrotising fasciitis secondary to soft tissue damage by bacteria and digestive juices [7, 8].

The ECF aetiology is the single best prognostic factor and predictor of therapeutic success [9]. In fact, the cause and underlying abnormalities strongly influence the likelihood of spontaneous closure and dictate the need for additional medical, surgical, or interventional treatment; therefore, the following sections will present the postoperative, CD-related and malignant ECFs separately with their specific imaging features and therapeutic approaches [1–3, 6].

## Post-surgical enterocutaneous fistulas

### Clinical features

Despite improved techniques, most ECFs currently develop after surgical procedures, secondary to anastomotic leakage, disruption of repaired enterotomy, or inadvertent intraoperative bowel injury. Some series reported an alarming incidence (0.8–2%) of postsurgical ECFs, probably resulting from the increasing complexity of procedures such as cytoreductive surgery with peritonectomy. Currently, the majority of ECFs are encountered in patients operated for colorectal and ovarian cancers. Alternatively, ECFs may develop following non-oncologic surgery such as ventral hernioplasty using prosthetic mesh, or repeated laparotomies such as for adhesiolysis and recurrent bowel obstruction. In our experience, the majority of patients had complex histories including multiple surgical interventions [4, 10–12].

Post-surgical ECFs may manifest early (within the first postoperative week) with a combination of septic fever, tachycardia, and hypotension, respiratory distress, progressively distended bowel, localised or diffuse abdominal tenderness, acidosis, leukocytosis, and elevated C-reactive protein. Alternatively, ECFs may present late during prolonged postoperative hospitalisation or after discharge as a tender, erythematous, fluctuant area at the surgical incision, or laparoscopic port site; in such cases nurses often notice medication gauzes becoming wet with either bile, enteric material, or stool [2].

### Radiographic fistulography: technique and appearances

**Fig. 1:**
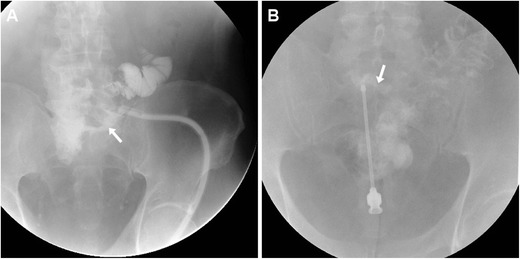
Two fistulography examples. **A** Fistulography performed via a catheter piercing through the external fistulous opening (EFO) in a 74-year-old man with history of repeated surgeries to manage bowel obstruction, adhesions and anastomotic dehiscence: optimal visualisation of course and length of the enterocutaneous fistula (ECF, arrow), in communication with the ileum. **B** Fistulography using a needle in a 60-year-old HIV-positive man with recent acute colonic diverticulitis: the ECF (arrow) communicating with the sigmoid colon required left hemicolectomy and ECF debridement

Fistulography (sinography) traditionally represented the mainstay technique to investigate ECFs and currently remains useful as a complementary technique to confirm communication between the EFO and a segment of the gastrointestinal tract. In our experience, this technique is best performed with help from the referring surgeon, who probes the EFO and enters the ECF. Depending on its calibre, a venous cannula or angiographic needle, a standard syringe, a Foley-type catheter, a paediatric or nasogastric feeding tube may be used. Then, non-forceful manual injection of low-osmolar water-soluble iodinated contrast medium (CM) is performed under fluoroscopic observation. Inexpensive, rapid, and easy to perform, fistulography directly visualises the communicating small bowel (Fig. 1A, B) or colonic segment (Fig. 1C) and provides information on ECF anatomy such as calibre, length, course, and ramifications. False negative studies may result from oedema, debris, or compression by abscess or mass impeding CM flow into the intestinal lumen. However, fistulography lacks cross-sectional visualisation of mural and extraluminal pathology upstream, downstream, and at site of the ECF [4, 5].

### CT techniques, appearances, and differential diagnoses

**Fig. 2:**
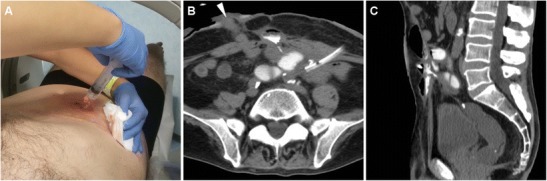
CT-fistulography technique, directly performed on the scanner table by manual injection through the EFO of diluted CM using a syringe and a venous cannula (**A**). An example of CT-fistulography (**B**, **C**) in a 69-year-old man with ulcerative colitis, previous right hemicolectomy and repeated surgery for complications: shortly after ileostomy closure (note drainage tube) low-output ECF was diagnosed, with communication between the EFO (arrowhead in **B**) and the opacified ileum

Nowadays the vast majority of patients should undergo CT or MRI studies to provide comprehensive cross-sectional information on ECFs and underlying disorders as a consistent basis for appropriate treatment choice and planning. Multidetector CT is preferred in uncooperative or severely ill patients such as those in early postoperative hospitalisation, because it obtains high-spatial resolution images in seconds, thus limiting motion or peristalsis artefacts. In the setting of ECF, intravenous CM is warranted unless contraindicated. Orally administered CM opacification may be helpful in patients with scarce intra-abdominal fat, to ease differentiation of bowel loops from extraluminal structures and abnormal collections; unfortunately, most patients who recently underwent surgery have ileus or obstruction and are unable to drink diluted CM. Alternatively, CT-fistulography (Fig. 2) with prior injection of iodinated CM such as 3% diluted iopamidol or iohexol through the EFO represents a useful one-stop-shop technique, which combines cross-sectional information with opacification of involved bowel tract. Image reconstruction and study interpretation along the sagittal plane are strongly recommended, as it provides the best visualisation of the ventral abdominal wall [4, 5].

**Fig. 3:**
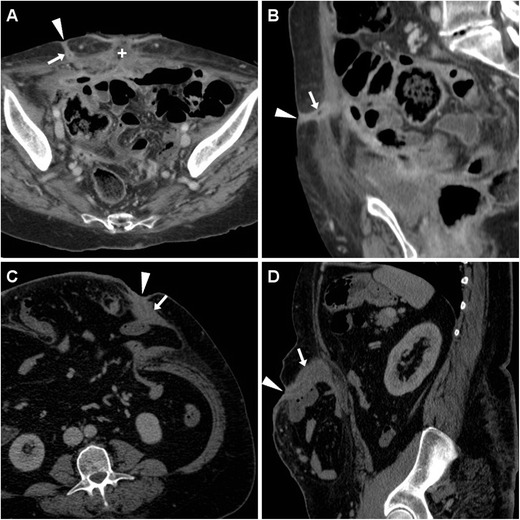
Early postoperative ECF in a 74-year-old woman following cytoreductive surgery for advanced endometrial carcinoma with peritoneal carcinomatosis. Characteristic CT (**A**, **B**) findings included enhancing “tram-track” paired structures (arrows) crossing from the peritoneal cavity through the anterior abdominal wall to the EFO (arrowheads), separated from the recent laparotomic access site (+). The ECF ultimately healed with prolonged in-hospital conservative treatment Another ECF developing in a 54-year-old obese woman with post-incisional abdominal evisceration: corresponding CT (**C**, **D**) appearance included thick-walled enhancing fistula (arrows) coursing from a herniated small bowel loop to the focally retracted EFO (arrowheads). Surgical treatment included ECF debridement and mesh hernioplasty

**Fig. 4:**
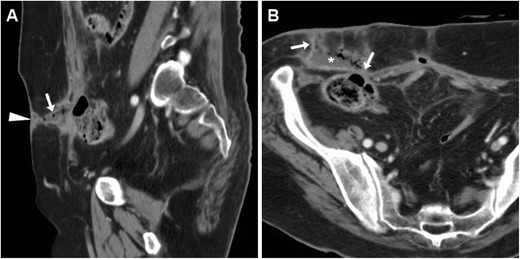
Postoperative ECF with subcutaneous abscess formation in a 71-year-old woman, following resection of sigmoid colon diverticulitis, complicated by anastomotic dehiscence which required re-laparotomy, ileal resection, and temporary ileostomy. CT diagnosed communication (arrows) between the cecum and an abscess (*) located just superficially to the ventral abdominal muscles, draining to the EFO (arrowhead in **A**). Surgical treatment included ileocecal resection and abscess toilette

The usual CT appearance of an ECF (Fig. 3) includes a tubular structure originating from a bowel loop, which directs ventrally or ventro-laterally through the peritoneum, crosses the abdominal wall muscles and fasciae to reach the cutaneous surface. ECFs may appear either collapsed or patent with gaseous and/or fluid content, in the latter case resulting in the characteristic “tram-track” appearance. Fistulous walls may be more or less thick, sometimes measuring up to one centimetre (Fig. 3C, D). The formation of a peripherally enhancing abscess with fluid and gaseous content along the ECF track (Fig. 4) should be reported, since it generally modifies the therapeutic approach.

**Fig. 5:**
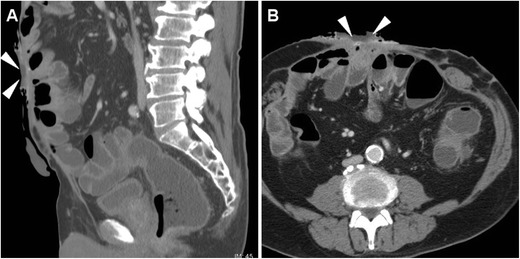
High-output ECF in a 69-year-old man with history of repeated surgeries to manage jejunal and duodenal perforations from Zollinger-Ellison syndrome, followed by hemicolectomy, ileostomy, and colostomy because of ischemic bowel necrosis. At CT, the wide draining EFO corresponded to a 2-cm hypoattenuating discontinuity (arrowheads) in the thickened skin, closely adherent to distended bowel loops with air-fluid levels consistent with postoperative ileus

In our experience, postsurgical ECFs often develop in cachectic patients with very thin subcutaneous and muscular planes of the ventral abdomen following repeated surgeries. The involved bowel loops tend to converge towards the ECF site and adhere closely to the peritoneal serosa (Figs. 2 and 5); not unusually, an ECF develops within a post-incisional hernia (Fig.3C, D).

At CT, the EFO generally corresponds to a focal cutaneous retraction or depression, and is often surrounded by thickened inflamed skin (Figs. 3 and 4); alternatively, a frank cutaneous breach or discontinuity may be observed, particularly in high-output ECFs (Fig. 5).

**Fig. 6:**
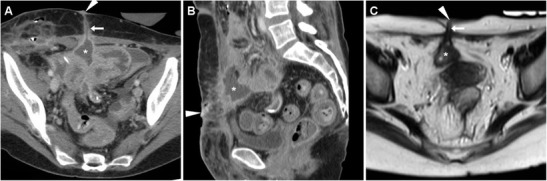
Two examples of postsurgical fistulas draining intra-abdominal collections, without bowel involvement. In a 34-year-old woman, after ileocecal resection CT (**A**, **B**) showed an EFO (arrowheads) communicating via a “tram-track” fistula (arrows) through the abdominal wall with a peripherally enhancing collection (*) which ultimately resolved. In a 48-year-old woman with recent CD resection, T2-weighted MRI (**C**) depicted a chronic postsurgical collection (*) connected to the skin by a fistula (arrows)

The two key CT differential diagnoses of an ECF are represented by:

- A recent laparotomic incision (Fig. 3A), which is differentiated by reviewing the surgical description and inspecting the patient’s abdomen, and
- Those fistulas (with usual “tram-track” CT appearance), which drain externally an intra-abdominal postsurgical collection (Fig. 6A, B), but lack communication with the bowel.

### MRI technique and findings

Borrowing from extensive experience with perianal fistulas, MRI has intrinsically superior soft tissue contrast compared to CT and may, therefore, better define the fistulous tract; furthermore, MRI is appealing to both radiologists and patients because of absent ionising radiation exposure. However, in critically ill patients such as those in the early postoperative days, MRI is hampered by respiratory and peristaltic artefacts from bowel obstruction or ascites. In patients who can sufficiently cooperate, MRI protocols to investigate ECFs should rely on multiplanar fluid-sensitive T2-weighted images including the anterior abdominal wall; the use of fat suppression techniques in at least one plane is recommended to improve detection of oedema and inflammation in the intra-abdominal and subcutaneous fat. Additionally, intravenous administration of gadolinium CM is helpful to visualise hypervascularity of ECF walls, abscess collections and surrounding fat planes, which is best appreciated on volumetric fat-suppressed T1-weighted gradient-echo sequences such as T1 high-resolution isotropic volume excitation (THRIVE), liver acquisition with volume acquisition (LAVA) or volumetric interpolated breath-hold examination (VIBE).

**Fig. 7:**
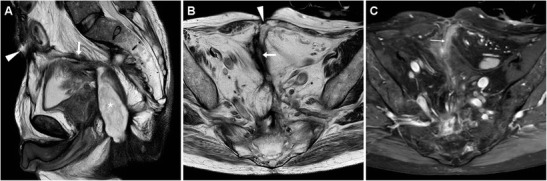
MRI diagnosis of post-surgical ECF in a 65-year-old male with long-standing fistulising CD, previous abdomino-perineal resection for perianal fistulas complicated by anal squamocellular carcinoma (ASC). On T2-weighted images, the fluid-filled ECF (arrows) was seen coursing ventrally to reach the ventral EFO (arrowheads) at the site of past surgical incision, from the apex of a large T2-hyperintense mass (* in **A**) corresponding to recurrent ASC. Post-gadolinium fat-suppressed T1-weighted image (**C**) showed mucosal hyperenhancement (thin arrow) along the fistula track

On heavily T2-weighted MRI sequences, the ECF is seen as a fluid-filled hyperintense tubular structure coursing through the abdominal wall and subcutaneous fat to reach the EFO. The fistulous walls show variable, generally intermediate signal intensity on both T1- and T2-weighted acquisitions, with corresponding “tram-track” enhancement (Fig. 7) on post-gadolinium images. Over time, persistent ECFs tend to develop thicker walls with lower T2-weighted signal from fibrosis, and decreasing intensity of contrast enhancement. Unfortunately, in our experience identification of the presence or absence of bowel communication is generally more challenging at MRI (Fig. 6C, D) compared to CT [4, 5].

### Treatment

Currently, the majority of postoperative ECFs tend to heal with conservative measures, endoscopic or percutaneous therapies. The latter consist in local injection of sealants such as cyanoacrylic glue (Glubran 2, GEM, Viareggio, Italy), and is rapidly becoming the preferred option as it achieves therapeutic success in a single session in almost two-thirds of cases, without any complications [5, 13].

Endoscopic positioning of clips and stents is the mainstay option when a leaking bowel anastomosis is present. Surgical repair is nowadays reserved for selected cases and remains associated with significant morbidity; high expertise is required to perform adhesiolysis and bowel resection at the internal fistula opening; unfortunately, postoperative ECF recurrence is not uncommon (16–21% of cases) [3, 14].

## Enterocutaneous fistulas in chronic inflammatory bowel diseases

### Clinical features

In patients with IBD, enterocutaneous fistulas may be either spontaneous or postoperative. The former situation results from the characteristic transmural inflammation of the affected enteric wall, which leads to penetration into the adjacent tissues, sometimes involves the abdominal wall and may ultimately reach the superficial tissues. In the vast majority of cases, ECFs arise from the diseased distal ileum and are associated with long-standing or active fistulising CD; exceptionally, an ECF may be the initial manifestation of an undiagnosed IBD. Albeit uncommon, CD still represents the main cause of non-iatrogenic ECFs and should therefore be suspected in a non-healing spontaneous fistula in a young or middle-aged, otherwise healthy patient [15, 16].

### Cross-sectional imaging

According to the European Crohn’s and Colitis Organisation (ECCO) statements, CT- and MR-enterography with oral luminal distension represent the ideal techniques for imaging diagnosis and follow-up of CD, with similar indications and diagnostic accuracy; the latter modality is increasingly used since it does not use ionising radiation. In selected patients, water-enema multidetector CT may be useful to visualise the extent, mural and extraluminal changes of colonic IBD. In urgent conditions, standard contrast-enhanced CT is widely used and highly helpful when acute CD complications such as perforation, obstruction, fistula, or abscess formation are suspected [17, 18].

**Fig. 8:**
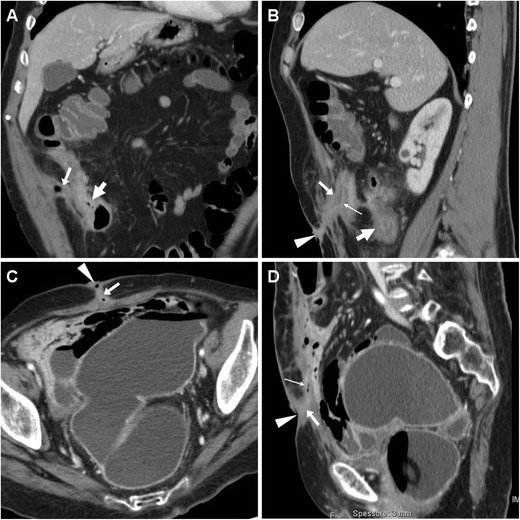
Two cases of spontaneous, low-output ECF in CD. In a 59-year-old man with a history of ileocecal resection nearly 20 years earlier, CT (**A**, **B**) identified a right-sided ECF as an enhancing “tram-track” fistula (arrows) with mucosal hyperenhancement (thin arrow), which departed from the thickened preanastomotic ileum (thick arrow) with recurrent CD and crossed the abdominal wall and subcutaneous fat to the EFO (arrowheads); repeated ileocolonic resection and ECF debridement were performed In a 62-year-old woman with several surgeries, water-enema CT (**C**, **D** - note distended rectosigmoid colon with rectal tube) showed a paramedian hypogastric ECF with mucosal hyperenhancement (thin arrow) and focally depressed EFO (arrowheads), communicating with the collapsed ileum; the patient did well on infliximab therapy

**Fig. 9:**
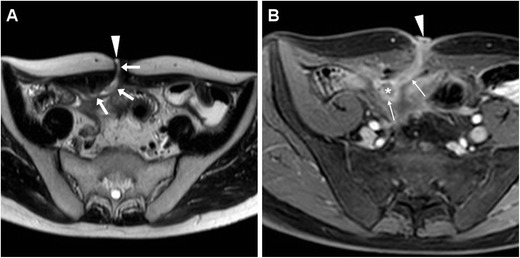
Spontaneous ECF in a 37-year-old man with stricturing and fistulising CD, shown at MR-enterography as a fluid-like T2-hyperintense track (arrows in **A**) coursing from an ileal loop through the abdominal wall, and finally reaching the EFO (arrowheads). Post-gadolinium fat-suppressed T1-weighted acquisition (**B**) showed mucosal hyperenhancement along ECF track (thin arrows) and at site of internal opening (*). Surgical treatment required fistula debridement plus stricturoplasty

**Fig. 10:**
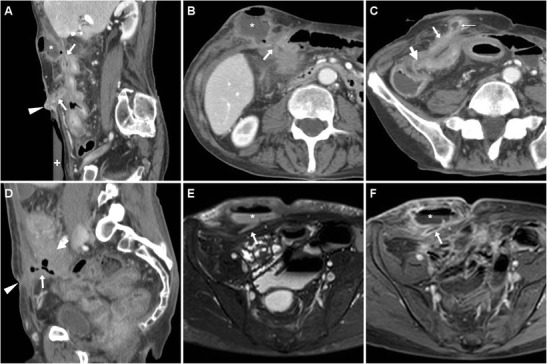
Two cases of spontaneous ECF complicated by abscess collections in chronic inflammatory bowel diseases. A 77-year-old man with indeterminate colitis treated by proctocolectomy and definitive ileostomy developed two ECFs, one in right hypochondrium with a sizeable superficial abscess (* in **A**, **B**) communicating (arrows) with a jejunal loop; the second, longer and more distal ECF (arrow in **C**) originated from the thickened ileum (thick arrow) and included a smaller subcutaneous abscess with inflamed mucosa (thin arrow). In a 59-year-old woman with fistulising CD, the ECF corresponded at CT (**D**) to a gas-filled track (arrow) arising from the diseased ileocolonic anastomosis (thick arrow) and reaching a subtle depressed EFO (arrowhead). Follow-up MRI including T2- (**E**) and post-contrast fat-saturated T1-weighted (**F**) showed development of a large abscess (*) with fluid content and enhancing wall along the ECF (arrows); definitive surgical treatment required extensive adhesiolysis, resection of rectum and of diseased perianastomotic bowel

Particularly in patients with penetrating CD, the anterior abdominal wall should be carefully scrutinised on cross-sectional imaging studies. Sometimes unexpected or subtle, ECFs are recognised at CT as “tram-track” structures with fluid and/or gaseous content (Fig. 8), which generally depart from the site of active disease, entero-enteric fistulas, or anastomotic CD recurrence. With MRI, fluid-filled ECFs are well recognised along their course from the bowel to the skin (Fig. 9). In the setting of active IBD, a characteristic appearance is the mucosal contrast enhancement, which is generally prominent and appreciated on either CT (Fig. 8) and on fat-suppressed MRI sequences (Figs. 9 and 10). More common than with post-surgical ECFs, abscess formation is characteristic of CD: with both modalities, abscess collections with peripheral enhancement, and mixed air, fluid and dense content are easily detected at either internal opening or along the subcutaneous track (Fig. 10) [19, 20].

### Post-surgical enterocutaneous fistulas in CD

Alternatively, an ECF may occur within a few weeks or months after IBD surgery, often through a previous abdominal scar and without residual or recurrent disease; clinically, this situation is commonly associated with limited physical findings due to use of steroids or immunomodulators.

**Fig. 11:**
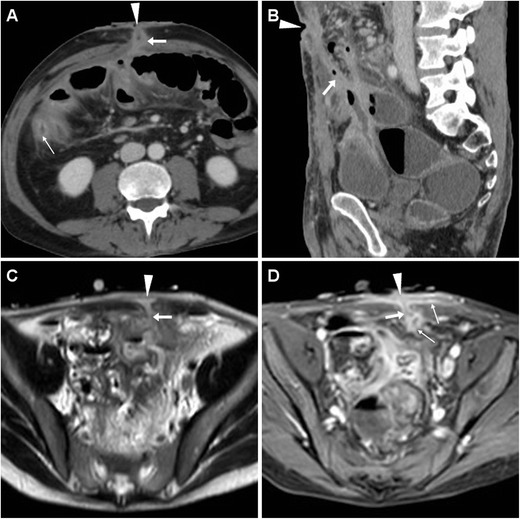
Two cases of post-surgical ECF in CD. After recent ileocecal resection, in a 61-year-old man multiplanar CT-enterography (**A**, **B**) showed mural thickening with mucosal hyperenhancement (thin arrows) along distal ileum consistent with disease activity, and an ECF (arrows) coursing obliquely from the affected tract to small-sized mesogastric EFO (arrowheads); reoperation included ECF debridement, ileal resection and redo anastomosis. A 27-year-old man developed an ECF after subtotal colectomy with cecal-rectum anastomosis, with corresponding MRI (T2-weighted image **C**, fat-suppressed post-contrast T1-weighted image **D**) appearance of fluid-filled track (arrows) with peripheral enhancement (thin arrows). Prominent inflammation also affected the skin surrounding the EFO (arrowheads) and at inner aspect of the ECF: the latter corresponded endoscopically to the anastomotic site and was treated by positioning of an over-the-scope clip, allowing ECF resolution

In patients who received surgery recently, multidetector CT (Fig. 11A–C) is preferred to confirm and visualise ECFs, with similar imaging findings to their spontaneous counterparts. Unfortunately, interpretation of cross-sectional imaging studies shortly after IBD surgery is generally difficult due to a combination of limited intra-abdominal fat and of postoperative changes such as diffuse oedema at resection site and in the mesentery, peritoneal fluid, and CM enhancement; in our experience this is particularly true with MRI (Fig. 11D–F) [15, 16].

### Treatment

The optimal treatment of enterocutaneous fistulas in CD remains debated: in the past, surgical resection of the diseased bowel plus fistula debridement through abdominal wall and subcutaneous planes was generally performed. In the last decade, medical therapy changed with introduction of biologics and immunomodulators. Following experience with perianal inflammatory disease, monoclonal antibodies against tumour necrosis factor are increasingly used: with correct patient selection, in the absence of stenosis and complex fistulas, infliximab achieves good response in postoperative and, to a minor extent, spontaneous ECFs too [16, 21, 22].

### Special situation: malignant fistulas in Crohn’s disease

In patients with long-standing CD, small bowel adenocarcinoma (SBAC) occurs 33 times more commonly than in the general population. Unfortunately, its unspecific manifestations mimic those of active or obstructive CD, resulting in frequently delayed diagnosis at an advanced stage with disseminated disease [23, 24].

**Fig. 12:**
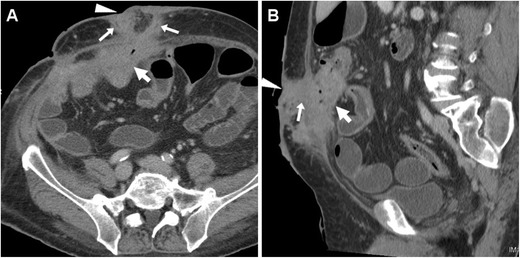
Ileal adenocarcinoma fistulising to the skin in a 68-year-old man with long-standing CD, previous ileocecal resection, recent weight loss and two draining EFOs at the anterior abdominal wall. CT-enterography confirmed double ventral ECF (arrows) from a markedly thickened non-stratified ileal segment (thick arrows). Neoplastic infiltration of the anterior abdominal wall was pathologically confirmed after en-bloc surgical resection. Tumour progression with ascites and liver metastases (not shown) ultimately developed (Partially reproduced from Open Access ref. no [34])

Sometimes, SBAC may penetrate through the anterior abdominal wall and fistulise to the skin (Fig. 12), a situation that is easily misinterpreted as the usual, spontaneous CD-related ECF unless thorough imaging is obtained. Unfortunately, differentiation of SBAC from either acute inflammatory or fibrotic CD is challenging. The commonest cross-sectional imaging patterns include ileal mass, long stricture with heterogeneous submucosa, short severe stenosis with upstream bowel dilatation, irregular asymmetric circumferential thickening. Suspicious features include mural thickness > 1 cm, abrupt “shouldering” transition, lost mural stratification, soft-tissue attenuation or solid MRI signal intensity, irregular serosal nodularity, and adenopathies (Fig. 12) [25–27].

The rare possibility of SBAC should be considered in patients with worsening CD symptoms after long quiescence and in non-healing ECFs despite treatment. Albeit difficult, preoperative diagnosis of malignant ECF in CD impacts treatment since it requires en bloc resection plus chemotherapy [23, 24]. Alternatively, the development of squamous cell carcinoma in chronic CD-related ECF has been occasionally reported [28].

## Irradiation

Albeit most patients receiving abdominal or pelvic irradiation develop some degree of radiation enteritis, post-radiation ECF are increasingly rare and develop at least 4–6 months after radiotherapy. Unfortunately, irradiation further increases complexity of management, due to a combination of three factors: a) low likelihood of spontaneous closure; b) poor healing and high risk of dehiscence following surgery on irradiated bowel; c) delayed or impossible adjuvant or palliative therapies in presence of an ECF [4].

## Tumour-related enterocutaneous fistulas

The possibility of an underlying malignancy should be considered when faced with a spontaneous ECF in an adult or elderly patient without history of recent surgery or IBD. This situation is increasingly rare since most colorectal malignancies are currently diagnosed and treated at an early or preclinical stage, but are strongly associated (over 50% of cases) with disseminated disease, impaired quality of life and high 30-day mortality [29, 30].

**Fig. 13:**
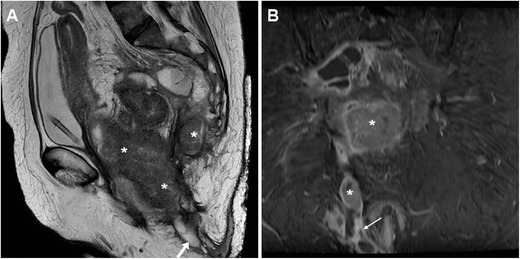
Locally advanced rectal cancer with superinfection and ECF formation in a 54-year-old woman with septic fever. T2-weighted MRI images (**A**) showed a large solid tumour (*) invading the vagina and perineum. Additionally, a 1-cm wide fluid-containing ECF (arrows) coursed postero-inferiorly towards the perianal skin and showed peripheral enhancement (thin arrow) on post-gadolinium fat-suppressed T1-weighted acquisition (**B**)

**Fig. 14:**
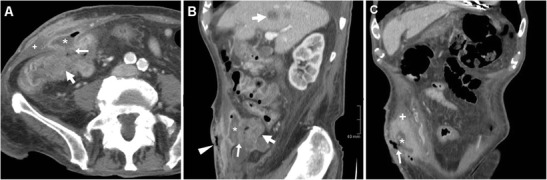
Cutaneous fistulisation of colon carcinoma in a 92-year-old man with worsening right-sided abdominal pain and tenderness. CT detected a segmental, non-stratified circumferential thickening (thick arrows) at the ascending colon, which infiltrated the peritoneum, opened (arrows in **A**, **B**) into an abscess (*) in the inflamed abdominal wall (+), and communicated to the skin EFO (arrowhead in **B**) through an ECF (arrow in **C**). Liver metastases (thick arrow) were present. The patient was treated supportively with positioning of colostomy-type bag at the EFO (Partially reproduced from Open Access ref. no [35])

Tumour-related ECFs result from either superinfection (Fig. 13) or fistulisation through the abdominal wall muscles (Fig. 14). In both cases, CT and MRI are crucial in identifying solid, more or less homogeneous and enhancing neoplastic masses (Fig. 13) and the possible presence of abscess collections requiring drainage. Alternatively, cross-sectional imaging suggests ECF-associated colorectal tumour when faced with irregular or asymmetric mural thickening (Fig. 14); features such as short segmental involvement, abrupt transition, lost mural stratification and lymphadenopathy further support a diagnosis of malignancy [31–33].

The ideal curative surgery is en-bloc removal of fistulising tumour along with the involved abdominal wall along the ECF track, plus derivative colostomy or ileostomy. Alternative options include palliative resection, bypass or stoma in unresectable tumours [10, 30].

## Conclusion

Nowadays, most patients with ECFs undergo multidetector CT or MRI studies to provide comprehensive cross-sectional assessment as a consistent basis for appropriate treatment choice and planning. In fact, visualisation of the underlying conditions (such as postoperative anastomotic leak, bowel obstruction, abscesses amenable to drainage, active or stricturing CD, fistulising or recurrent tumour) allows directed treatment, thus resulting in decreased rates of failed conservative management and higher likelihood of therapeutic success [4, 5].
